# A Mismatch Between Theory and Practice in the Transition From Home to a Nursing Home: A Scoping Review

**DOI:** 10.1093/geroni/igab046.857

**Published:** 2021-12-17

**Authors:** Lindsay Groenvynck, Amal Fakha, Bram de Boer, Jan Hamers, Matheus van Achterberg, Erik van Rossum, Hilde Verbeek

**Affiliations:** 1 Maastricht University, Maastricht, Limburg, Netherlands; 2 KU Leuven, Leuven, Vlaams-Brabant, Belgium; 3 Zuyd University of Applied Sciences, Heerlen, Limburg, Netherlands

## Abstract

The transition from home to a nursing home is a complex process, existing of three transition phases (pre-, mid- and post-transition). It is often fragmented, leading to negative outcomes for older persons and informal caregivers. To prevent these negative outcomes, knowledge of existing transitional care interventions is paramount. Therefore, a scoping review was performed, summarizing current interventions aiming to improve transitional care. The review identified 17 studies, describing eight multi- and five single-component interventions. From the multi-component interventions, seven main components were identified: education, relationships/communication, improving emotional well-being, personalized care, continuity of care, support provision, and ad hoc counseling. This review identified a clear mismatch between theory on optimal transitional care and current transitional care interventions. All interventions focused on either a specific phase or target population throughout the transition process. This inhibits a continuous transition process in which a partnership between all stakeholders involved exists.

